# Karrikins Regulate the Redox Balance and Sugar Metabolism of Postharvest Kiwifruit (*Actinidia deliciosa*)

**DOI:** 10.3390/plants14162567

**Published:** 2025-08-18

**Authors:** Mingxia Shao, Hongli Li, Shuhua Zhu, Dandan Huang, Chengkun Li

**Affiliations:** 1 College of Chemistry and Materials Science, Shandong Agricultural University, Taian 271018, China; smx623629@163.com (M.S.); lihongli07@126.com (H.L.); 2 Key Laboratory of Special Fruits and Vegetables Cultivation Physiology and Germplasm Resources Utilization, College of Agriculture, Shihezi University, Shihezi 832000, China

**Keywords:** karrikin, kiwifruit, reactive oxygen species, soluble sugars metabolism, ascorbate, glutathione

## Abstract

Karrikins, a class of butenolide compounds derived from plant-derived smoke, positively regulate plant development and stress tolerance. However, their effects on postharvest fruit have scarcely been reported. In this study, karrikin solution was prepared by absorbing maize straw smoke into water, and kiwifruits (*Actinidia deliciosa*) were immersed in different concentrations of this solution to determine the optimal concentration based on respiratory rate, relative conductivity, firmness, soluble solids content, and appearance of the kiwifruits. Subsequently, the regulation of reactive oxygen species (ROS) and soluble sugars metabolism by karrikins were studied. The results showed that the optimal dose of karrikins for kiwifruit was 1.20 μmol L^−1^. Karrikins enhanced the activities of superoxide dismutase, catalase, enzymes in the ascorbate–glutathione pathway, and soluble sugars metabolism, increased the concentrations of reducing ascorbate, glutathione, sucrose, and fructose-6-phosphate, suppressed ROS concentrations, and maintained the quality of kiwifruit during storage. These results suggest that karrikins could be a potential tool to modulate fruit ripening, with their effects depending on the dosage used.

## 1. Introduction

Kiwifruit (*Actinidia deliciosa*) is a highly nutritious and distinctive fruit; however, it possesses a limited shelf life and is susceptible to spoilage. Consequently, it is essential to use the correct storage method to maintain the freshness and quality of the kiwifruit. Various preservation techniques have been developed for kiwifruit, including low-temperature [1], controlled atmosphere storage [2], chemical approaches [3], and biological strategies [4]. Among these methods, preservatives such as 1-MCP (1-Methylcyclopropene) [5], nitric oxide [6], melatonin [7], plant essential oils [8], and other natural compounds [9] are commonly used to maintain the quality of postharvest kiwifruit [10].

Sugar concentrations critically influence fruit quality, drive ripening processes [11], and affect consumers’ acceptability of kiwifruit [12]. Sucrose, fructose, and glucose are the main sugars in kiwifruit [13]. Generally, sucrose is primarily synthesized from fructose-6-phosphate (F-6-P) in a reaction catalyzed by sucrose phosphate synthase (SPS), which is largely irreversible under physiological conditions. Sucrose metabolism also involves sucrose synthase (SS), which catalyzes a reversible reaction: SS can both synthesize sucrose from glucose and fructose or hydrolyze sucrose to glucose and fructose [14]. Thus, SS exhibits dual activities (synthesis and decomposition), with reaction directionality modulated by sucrose and fructose concentrations [15]. Alkaline invertase (AI) and neutral invertase (NI) catalyze the irreversible sucrose hydrolysis into glucose and fructose [16]. These soluble sugars modulate fruit quality dynamics during storage by relieving osmotic stress, maintaining energy for cell survival and reactive oxygen species (ROS) homeostasis [17].

Reactive oxygen species (ROS) serve as signaling molecules that regulate plant development and responses to stress. However, the biological functions of ROS depend on its dose [18]. During postharvest storage, excessive ROS accumulation leads to oxidative damage in fruit [19]. Maintaining redox balance is therefore critical for preserving fruit quality [20]. Antioxidative enzymes, including catalase (CAT), superoxide dismutase (SOD), peroxidase (POD), polyphenol oxidase (PPO), and the ascorbate–glutathione (AsA-GSH) cycle, contribute to maintaining the redox balance in fruit [19,20]. Postharvest techniques target antioxidative systems to retard ROS-induced oxidative stress, thereby maintaining redox balance, improving fruit quality [21], and synergistically alleviating kiwifruit senescence [22].

Karrikins (KARs), a type of butanolide compound found in smoke from burning or charring plants, have been confirmed to comprise six different structures (KAR_1_-KAR_6_), all of which contain a butenolide moiety fused with a differential methyl-substituted pyran ring [23]. The structural features of karrikins are similar to strigolactones, which suggest analogous plant signaling pathways [24]. Karrikins are perceived by the receptor KAI2 (Karrikin insensitive2), an α/β hydrolase that recognizes KARs as structural mimics of the putative KAI2 ligand phytohormone [25]. Furthermore, Karrikins regulate redox homeostasis and enhance plant responses to abiotic stress [26,27]. For instance, Karrikins have been demonstrated to enhance cold tolerance in tomatos [28], promote leaf expansion [29], and stimulate seed germination in plants [30]. The biological functions of karrikins are of great interest to researchers and have become a key area of focus in plant research. Although research on karrinkins’ biological functions has expanded rapidly, current studies primarily focus on the regulatory effects on plant development and stress adaptation. To our knowledge, there is no report on the regulation of the quality of fruit during storage by karrikins at present. This study aimed to investigate whether karrikins modulate the senescence of kiwifruit, focusing on the regulatory activity of karrikins on redox balance and soluble sugars metabolism to develop a novel preservation strategy for kiwifruit and a basis for the practical application of karrikins.

## 2. Results

### 2.1. Karrikins Extended Storage and Maintained Kiwifruit Quality

**Figure 1:**
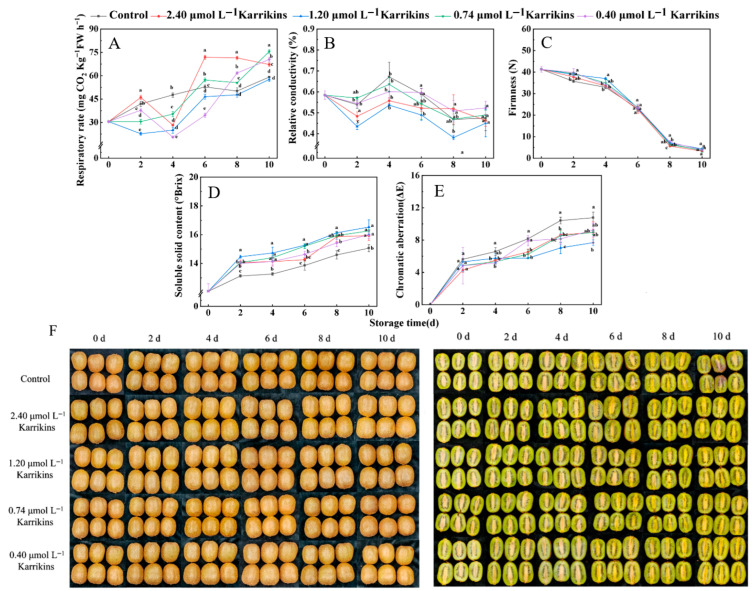
Changes in respiratory rate (**A**), relative conductivity (**B**), firmness (**C**), soluble solids content (**D**), chromatic aberration (**E**), and appearance of kiwifruits(*Actinidia deliciosa*) treated with different concentrations of karrikins (**F**). Different letters indicate significant differences (*p* < 0.05) between the control and karrikin treatments at the same time.

**Figure 2:**
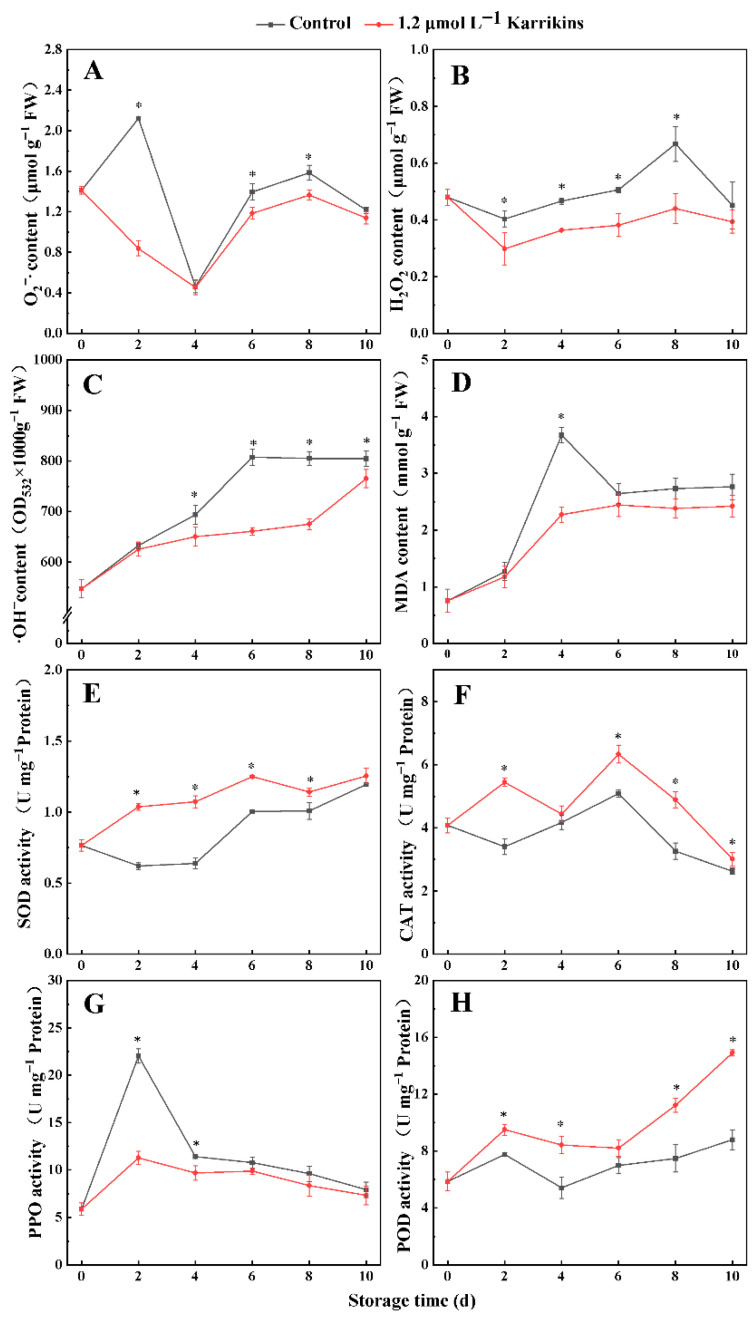
ROS, MDA concentrations, and antioxidant enzyme activity in kiwifruit treated with different concentrations of karrikins. O_2_^−•^ concentration (**A**); H_2_O_2_ concentration (**B**); •OH concentration (**C**); MDA concentration (**D**); SOD activity (**E**); CAT activity (**F**); PPO activity (**G**); POD activity (**H**). The asterisk indicates significant differences (*p* < 0.05) between the control and 1.20 μmol L^−1^ karrikin treatment at the same time. FW = fresh weight.

Karrikins’ effect on kiwifruit (*Actinidia deliciosa*) quality exhibited dose and time dependence (Figure 1). Except for 2.40 μmol L^−1^ karrikin treatment on day 2, the rest of the treatments with karrikins decreased the kiwifruit respiratory rate before day 4. However, respiration rates in kiwifruit treated with 2.40 and 0.74 μmol L^−1^ karrikins were higher than those of the control after day 6 (*p* < 0.05). Notably, 1.20 μmol L^−1^ karrikins maintained a lower respiratory rate throughout storage, despite non-significant differences versus controls on days 8 and 10 (Figure 2A). Karrikins maintained low relative conductivity in kiwifruit during storage. In particular, kiwifruit treated with 1.20 μmol L^−1^ karrikins exhibited the lowest relative conductivity compared to the control and other treatments (Figure 2B). Karrikins also maintained kiwifruit firmness on days 2 and 4, although there was no significant difference in firmness among the treatments and the control at the last storage stage (Figure 2C). However, 1.20 μmol L^−1^ karrikins significantly increased soluble solids content (SSC) versus controls and other concentrations (*p* < 0.05; Figure 2D). Meanwhile, kiwifruit treated with 1.20 μmol L^−1^ showed a minor color change and better appearance than those in other treatments (Figure 2E,F). Considering the above results, karrikins at 1.20 μmol L^−1^ was identified as the optimal dose for subsequent mechanistic studies.

### 2.2. Karrikins Decreased ROS Concentrations and Regulated Antioxidative Enzyme Activities

Treatment with 1.20 μmol L^−1^ karrikins maintained lower concentrations of O_2_^−•^, H_2_O_2_, and •OH in kiwifruit compared to the control during storage (Figure 2). In kiwifruit treated with karrikins, the O_2_^−•^ concentration on days 2, 6, and 8 was 40%, 85%, and 86% of the control, reducing H_2_O_2_ to 66% on day 8 and suppressing •OH formation to 84% on day 8. Notably, the MDA concentration showed a significantly lower (*p* < 0.05) level exclusively on day 4. Concurrently, treatment of 1.20 μmol L^−1^ karrikins enhanced antioxidant enzyme activities: SOD activity increased to 1.67 times that of the control on day 2 and 1.68 times on day 4; CAT activity rose to 1.60 times on day 2 and 1.24 times on day 6; and POD activity reached 1.70 times the control by day 10. In contrast, polyphenol oxidase (PPO) activity was potently inhibited to 51% of the control on day 2.

### 2.3. Karrikins Promoted the AsA-GSH Cycle in Kiwifruit

**Figure 3:**
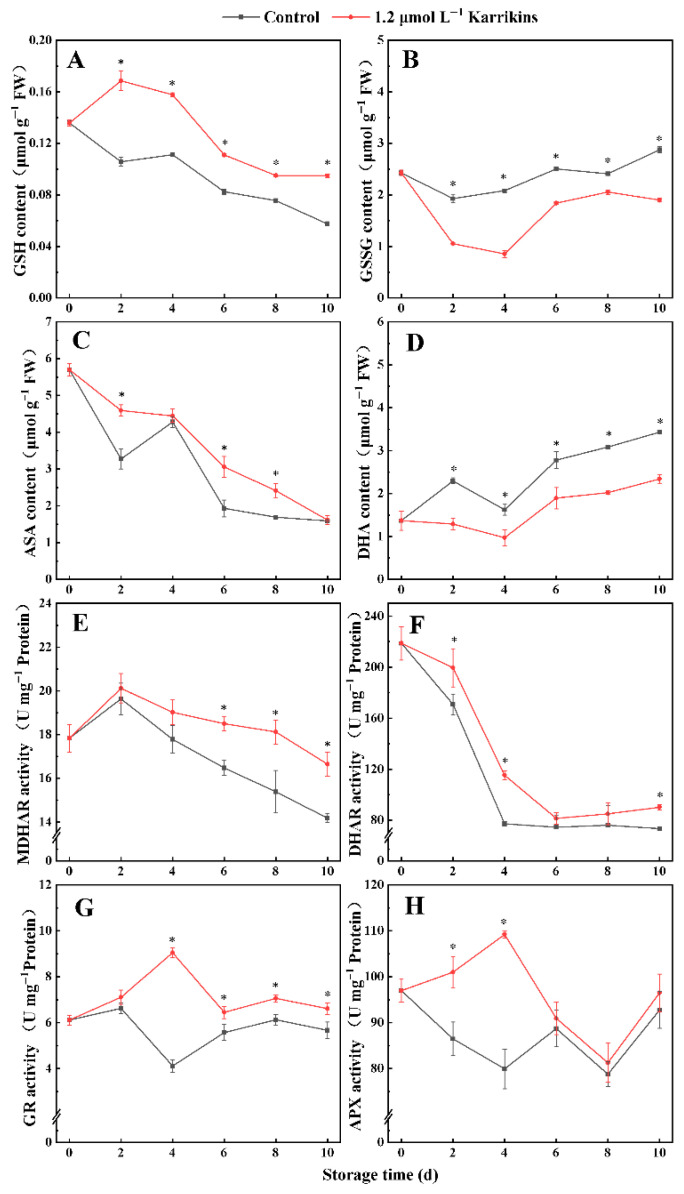
Substance concentrations and enzyme activities of the AsA-GSH cycle in kiwifruit after treatment with karrikins. GSH concentration (**A**); GSSG concentration (**B**); AsA concentration (**C**); DHA concentration (**D**); MDHAR activity (**E**); DHAR activity (**F**); GR activity (**G**); APX activity (**H**). The asterisk indicates significant differences (*p* < 0.05) between the control and 1.20 μmol L^−1^ karrikin treatment at the same time. FW = fresh weight.

Karrikins potently modulated the ascorbate–glutathione redox cycle in kiwifruit (Figure 3). Treatment with karrikins elevated GSH concentrations to 1.60-fold (day 2) and 1.42-fold (day 4) versus the control (Figure 3A), concomitantly with GSSG suppression to 55% (day 2) and 41% of the control (day 4) (Figure 3B). Concurrently, AsA concentration was improved to 1.40-fold and 1.58-fold of the control on days 2 and 6, respectively (Figure 3C), whereas DHA concentration on days 2 and 10 was reduced to 56% and 68% of the control (Figure 3D).

Karrikins at 1.20 μmol L^−1^ availably improved MDHAR, DHAR, GR, and APX activities in kiwifruit during storage. In karrikin-treated kiwifruit, MDHAR activity increased to 1.18, 1.17 times that of the control on days 8 and 10, DHAR activity rose to 1.49, 1.23 times that of the control on days 4 and 10, GR activity on day 4 was 2.20 times that of the control, and APX activity was elevated to 1.17, 1.37 times that of the control on days 2 and 4.

### 2.4. Karrikins Modulated Soluble Sugars Concentrations and Enzyme Activities Related to Sugar Metabolism

**Figure 4:**
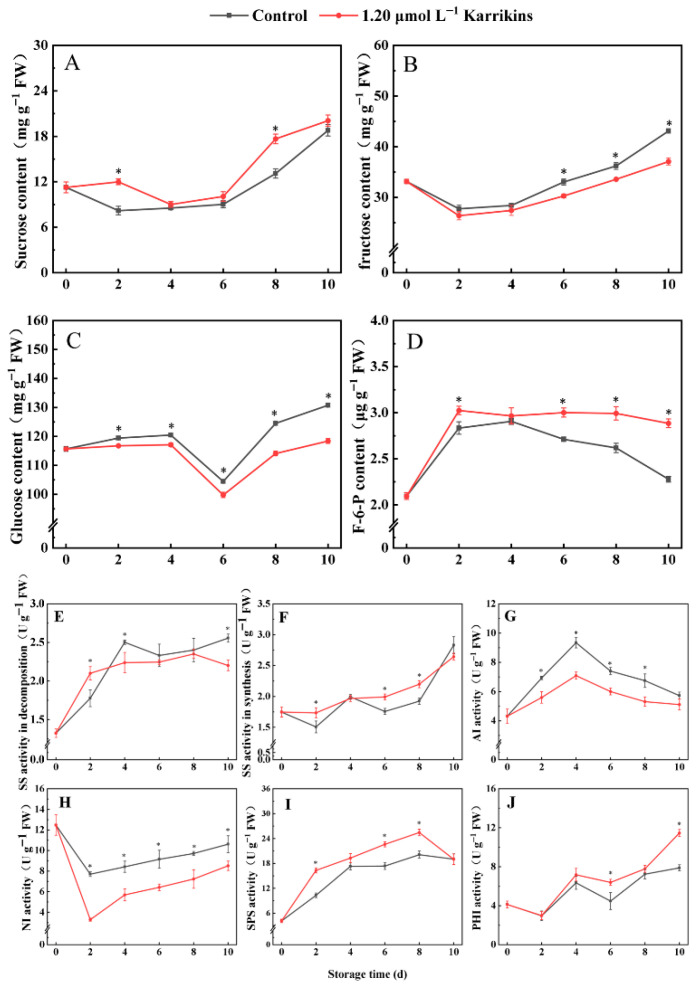
Soluble sugars concentrations and the activities of enzymes of sugar metabolism in kiwifruit treated with karrikins. Sucrose concentration (**A**); fructose concentration (**B**); glucose concentration (**C**); F-6-P concentration (**D**); SS activity in decomposition (**E**); SS activity in synthesis (**F**); AI activity (**G**); NI activity (**H**); SPS activity (**I**); PHI activity (**J**). The asterisk indicates significant differences (*p* < 0.05) between the control and 1.20 μmol L^−1^ karrikin treatment at the same time. FW = fresh weight.

As shown in Figure 4, kiwifruit treated with 1.20 μmol L^−1^ karrikins exhibited higher sucrose concentrations than the control on days 2 and 8 (*p* < 0.05), reaching 1.46 and 1.35 times the control, respectively. In contrast, karrikins decreased the fructose concentrations during late storage, with treated fruit attaining 92% of control levels after day 6. Glucose concentrations in kiwifruit treated with karrikins were consistently lower than the control throughout the entire storage period, declining to 91% of the control on day 10. However, F-6-P concentrations were maintained at higher levels in karrikin-treated fruit, peaking at 1.27 times the control on day 10.

SS activity in decomposition in kiwifruit treated with karrikins was 1.18 times that of the control on day 2 but decreased to 89% and 86% of the control on days 4 and 10, respectively (Figure 4E). Conversely, SS activity in synthesis increased to 1.15, 1.13, and 1.14 times the control on days 2, 6, and 8, respectively (Figure 4F). Karrikins inhibited AI and NI activities throughout storage; notably, AI activity was only 81% of the control on day 4 (Figure 4G), and NI activity was only 42% of the control on day 2 (Figure 4H). Additionally, karrikins enhanced SPS and PHI activities: SPS activity increased to 1.58, 1.30, and 1.27 times the control on days 2, 6, and 8, respectively (Figure 4I), while PHI activity increased to 1.43 and 1.08 times the control on days 6 and 8, respectively (Figure 4J).

## 3. Discussion

Kiwifruit (*Actinidia deliciosa*), as a characteristic climacteric fruit, is typically harvested at a physiologically mature but unripe stage to prolong the storage time. This is necessitated by its vigorous postharvest respiratory metabolism, which drives rapid ripening and softening processes. Crucially, within consumer sensory evaluations of kiwifruit, firmness serves as a primary sensory cue for assessing both perceived ripeness stage and immediate edibility; however, for kiwifruit postharvest storage preservation, maintaining firmness is essential to effectively prolong storage duration and sustain quality.

Karrikins are characterized by a fundamental structure consisting of a five-membered lactone ring fused to a six-membered pyran ring, exhibiting chemical similarity to strigolactones that contain a butyrolactone moiety. Karrikins and strigolactones have been established as regulators of plant growth processes [31,32]. For instance, karrikins enhanced cold tolerance in tomato plants by modulating the strigolactones and abscisic acid (ABA) signaling networks [28]. Additionally, they regulated plant growth through effects on ABA and cytokinin homeostasis [33]. Experimental evidence further indicated that karrikins modulate fruit quality during postharvest storage, demonstrating optimal efficacy at 1.20 μmol L^−1^ for kiwifruit. At this concentration, karrikins retarded the increase in respiratory rate, reduced relative conductivity, and maintained the firmness, color, and soluble solids content of kiwifruit at proper levels, simultaneously regulating the ROS and soluble sugars metabolism in kiwifruit, exhibiting the potential positive roles of karrikins in regulating fruit quality during storage.

Respiratory rate is an essential indicator for climacteric kiwifruit, which peaks quickly during ripening [34]. Reducing the respiratory rate is a commendable method to maintain the quality and prolong the storage life of climacteric fruit. Karrikins at 1.20 μmol L^−1^ attenuated the respiratory rate in kiwifruit, thereby delaying firmness loss and suppressing ROS generation, which collectively contributed to extending fruit storage. Similar preservatives such as nitric oxide [6], hydrogen sulfide [35], and melatonin [36] also inhibited the respiration of kiwifruit during storage. However, karrikins can activate and enhance seed respiration to stimulate dormancy break [30]. These results showed the multiple functions of karrikins. The differential regulation by karrikins of retarding respiration in postharvest fruit but improving the respiration of dormancy seeds might depend on the plant species, plant tissues, karrikins dose, and so on.

**Figure 5:**
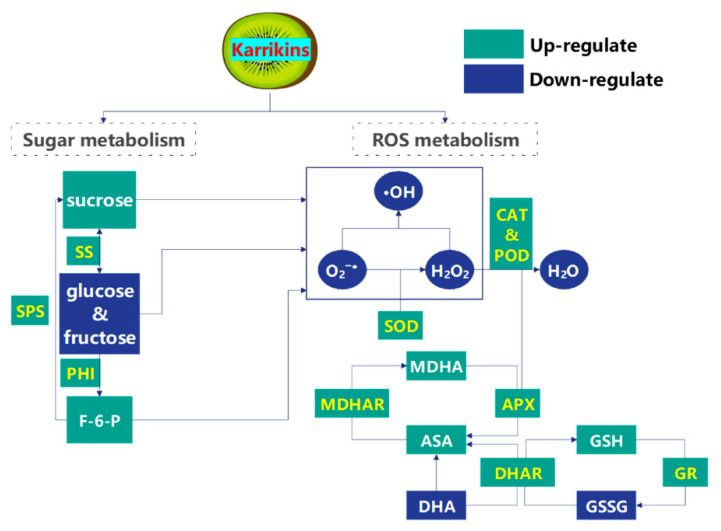
Possible regulation by karrikins of sugar metabolism and ROS homeostasis in kiwifruits.

ROS metabolism is integral to the ripening process and storage of fleshy fruit and protects fruit against pathogens at low concentrations but triggers oxidative damage at high levels [18]. Thus, ROS homeostasis is essential for maintaining normal physio-biochemical processes in fruit [20]. When postharvest fruit suffers from adversity stress, ROS bursts induce oxidative damage; consequently, the antioxidant systems are enhanced to restore ROS homeostasis [19]. In kiwifruit, karrikins reduced the accumulation concentrations of O_2_^−•^, H_2_O_2_, and •OH during storage, thereby preserving cellular membrane integrity through attenuation of electrolyte leakage. Similar changes in ROS concentrations were also found in kiwifruit treated with nitric oxide [37], ozone [38], and phytosulfokine α [39]. The enzymatic defense system (including SOD, CAT, POD, MDHAR, DHAR, GR, and APX) and the non-enzymatic defense system (including AsA, GSH, and phenolic substances) cooperatively counteract the oxidative stress caused by excessive ROS [19]. Karrikins maintained high activities of SOD, CAT, and POD while restraining PPO activity, which promoted the conversion from O_2_^−•^, H_2_O_2_, and •OH to H_2_O and O_2_, along with reducing the increase in chromatic aberration [19]. Meanwhile, the enhanced MDHAR, DHAR, GR, and APX activities within the AsA-GSH cycle promoted high concentrations of AsA and GSH, directly reducing ROS and alleviating oxidative damage in karrikin-treated kiwifruit (Figure 5). Similar results were also found in kiwifruit treated with p-coumaric acid [40] and 1-methylcyclopropene [41].

Karrikins can augment the total soluble sugars content of *Brassica alboglabra* and seeds of *Coriandrum sativum* under Cd and temperature stress [42,43]. However, no reports have demonstrated karrikins’ regulation of soluble sugars metabolism in postharvest fruit, given that soluble sugars content is a vital quality indicator and plays a crucial role in mitigating oxidative stress in response to abiotic stress [44,45]. In kiwifruit, karrikins increased the activities of SS in synthesis, SPS, and PHI while suppressing the activities of SS in decomposition, AI, and NI, resulting in increases in sucrose and F-6-P concentrations and decreased fructose and glucose concentrations, which suggested that exogenous karrikins could modulate carbohydrate metabolism to preserve higher soluble solids content and storage quality (Figure 5). On the other side, soluble sugars, such as glucose and sucrose, also function as antioxidants [45]. Sucrose at high concentrations can function as an antioxidative compound, unequivocally neutralizing ROS, inhibiting lipid peroxidation [38], and stimulating AsA biosynthesis in postharvest fruit [46]. Karrikins improved sucrose and AsA concentrations and decreased ROS and MDA concentrations in kiwifruit, thereby alleviating oxidative damage through a non-enzymatic defense system. Glucose, fructose, and F-6-P also exhibit high capacities for scavenging superoxide and modulating ROS homeostasis in plants [47,48]. Karrikin-treated kiwifruit showed a high concentration of F-6-P, indicating their potential capacity to scavenge excessive ROS. However, karrikins decreased the glucose and fructose concentrations in kiwifruit, mechanistically linked to upregulated enzymatic conversion of these hexose substrates to sucrose mediated by SPS and synthesis-oriented SS catalysis. Another reason for the lower concentrations of fructose and glucose in kiwifruit was that both sugars contain tautomerizable aldehydic groups, which are susceptible to oxidation by excessive ROS to form carboxylic acids or other compounds [49,50]. Additionally, the autoxidation of glucose and fructose constitutes an endogenous ROS source that promotes lipid peroxidation. Low glucose and fructose concentrations might reduce ROS production via sugar autoxidation, which might also be a possible reason why kiwifruits treated with karrikins had low ROS concentrations.

The impacts of karrikins on plant responses to abiotic stress have been widely confirmed [26]. However, karrikins’ regulatory roles in postharvest fruit, which similarly endure storage-induced abiotic stress, remain unexplored. Therefore, the regulation by karrikins on postharvest fruit was preliminarily investigated, and the results confirmed its positive role in improving the abiotic stress resistance of plants. The chemical structures and components of karrikins have been partially disclosed [51]; however, the smoke-water-derived karrikins used in this study contain complex maize combustion products, though it is a common method. Whether different karrikins components have similar or distinct roles in regulating plant stress resistance, and how they perform these roles in plants, remains unknown. The preliminary influence of karrikins on soluble sugars and ROS homeostasis in kiwifruit has been reported herein, and further research should explore how karrikins regulate soluble sugars metabolism to affect fruit quality and alleviate oxidative damage in fruit during storage.

## 4. Materials and Methods

### 4.1. Plant Materials

Karrikin solution was prepared according to the method of Liu et al. [28]. Briefly, maize straw (20 g) was placed in a stainless-steel basin and heated at 200 °C for approximately 10 min using an electric hot plate to produce smoke. The smoke was pumped into a vacuum bottle containing 4 L of water under vacuum for approximately 40 min until the straw became entirely blackened and no further smoke was generated. The insoluble matter was removed using a Büchner funnel (Beijing Synthware Glass, Inc., Beijing, China), and the filtrate was the karrikin solution. The concentration of karrikins was quantified at approximately 2.40 μmol L^−1^ by means of ultra-high-performance liquid chromatography–tandem mass spectrometry [52]. This karrikin solution was diluted with deionized water to final concentrations of 1.20, 0.74, and 0.40 μmol L^−1^, respectively.

‘Xuxiang’ kiwifruits (*Actinidia deliciosa*) were picked at 150 days after full bloom from a local orchard in Taian, China. Fruit with firmness of 41.0 ± 1.0 N and soluble solids content of 12.0 ± 1.0% without disease and mechanical damage were immersed in karrikin solutions (0.40, 0.74, 1.20, and 2.40 μmol L^−1^) for 20 min, respectively. The kiwifruit immersed in deionized water served as the control. Each treatment was performed with three independent biological replicates, and each replication contained 200 kiwifruits. After naturally drying, the fruits were stored at 25.0 ± 1.0 °C and 85 ± 5% relative humidity, with sampling at 2-day intervals [53].

Sample processing procedure: 16 fruits per treatment replicate were sampled daily for routine quality assessments, with 15 additional fruits processed through the aforementioned liquid nitrogen flash-freezing protocol for subsequent sugar and ROS metabolic analyses. Sampled fruits were immediately sectioned into small pieces, and the tissue fragments were then completely submerged in liquid nitrogen for instantaneous metabolic quenching. The flash-frozen samples were transferred to pre-chilled storage bags and maintained at −80 °C until analysis. Before metabolite extraction, the cryopreserved tissues were pulverized into fine powder using a cryogenic grinder (IKA Works (Guangzhou) Equipment Co., Ltd., Guangzhou, China). The resultant powder was immediately processed for metabolite extraction and subsequent quantification.

### 4.2. Measurement of Fruit Quality

The respiratory intensity of kiwifruit, expressed in mg CO_2_∙kg^−1^∙h^−1^, was determined using a SY-1022 gas analyzer (Shijiazhuang Shiya Technology Co., Ltd., Shijiazhuang, China) with the computational formula in the instruction book: respiratory intensity (CO_2_ mg kg^−1^ h^−1^) $=\frac{F\;\times c\;\times 60\times 10^{-3}\times 44}{W\;\times 22.4\times [273/(273+T)]}$, *F* (gas flow rate, mL∙min^−1^), *c* (volume fraction of CO_2_, μL∙L^−1^ ppm), *T* (°C), *W* (fruit quality, Kg), 44 (molar mass of CO_2_, g∙mol^−1^), 22.4 (L∙mol^−1^), 273 (K), 60 × 10^−3^ (time unit conversion factor). Relative conductivity (%) was determined using a DDS-307 conductivity meter (Jingke Leici Co., Shanghai, China) [54]. Fruit firmness in (N) was determined using a handheld fruit hardness tester (GY-4, Yueqing AideBao Co., Ltd., Wenzhou, China) fitted with a 3.5 mm probe. Soluble solids content (°Brix) was measured using a WY015R handheld digital refractometer (Nanjing Abes Co., Ltd., Nanjing, China). A CR-10 minolta colorimeter (Konica Minolta Sensing, Inc., Tokyo, Japan) was used to measure the L*, a*, and b* values of fresh-cut surfaces, and the color change (ΔE) was determined according to the rule of Magri et al. [55]:$\Delta E=\sqrt{{\left(L_{0}-L_{d}\right)}^{2}+{\left(a_{0}-a_{d}\right)}^{2}+{\left(b_{0}-b_{d}\right)}^{2}}$, ‘0’ denotes the initial value and ‘*d*’ denotes the values for different times of sampling.

### 4.3. Measurement of ROS Concentration and Antioxidative Enzyme Activities

Kiwifruit tissue was ground into powder using an A11 basic analytical grinder (IKA Works (Guangzhou) Equipment Co., Ltd., Guangzhou, China). H_2_O_2_, •OH concentrations (nmol g^−1^ FW) and O_2_^−•^ production rate (nmol min^−1^ g^−1^ FW) were determined according to the method of Yun et al. [56]. Kiwifruit (1 g) was homogenized with 3 mL of 100 g L^−1^ trichloroacetic acid, thoroughly extracted, and then centrifuged to obtain the supernatant for MDA (malondialdehyde) concentration determination, as described by Huang et al. [57].

POD activity was determined following the method of Jing et al. [58] via the guaiacol method, recording absorbance change at 470 nm. CAT activity was determined according to Maehly [59] by monitoring absorbance change at 240 nm. CAT and POD enzyme activities were defined as a decrease of 0.01 in absorbance value per unit of time corresponding to 1 unit of enzyme activity (U), expressed as U mg^−1^ protein. Polyphenol oxidase (PPO) activity was assayed according to the method of Rocha et al. [60]; the change of 0.001 in the absorbance at 420 nm was defined as 1 unit (U), and PPO activity was expressed as U mg^−1^ protein. Superoxide dismutase (SOD) activity was determined using the nitroblue tetrazolium (NBT) photoreaction method described by Vicente et al. [61]. The inhibition of NBT photoreduction by 1 unit (U) of SOD activity was 50%, and the results are expressed as U mg^−1^ protein.

Kiwifruit tissue (2 g) was mixed with 6 mL of 5% metaphosphoric acid and ground thoroughly. After centrifugation, the supernatant was collected. Oxidized ascorbic acid (DHA) concentration was calculated by subtracting the reduced ascorbic acid (AsA) from the total ascorbic acid [62]. The concentrations of oxidized glutathione (GSSG) and reduced glutathione (GSH) were extracted and quantified following the method outlined by Nagalakshmi et al. [63]. All concentrations are expressed as μmol g^−1^ FW.

Kiwifruit tissue (5 g) was combined with 5 mL of 50 mmol L^−1^ phosphate buffer (containing 0.1 mmol L^−1^ EDTA, 1 mmol L^−1^ ascorbic acid, and 2% PVPP) and mixed thoroughly at low temperature. The supernatant was extracted via centrifugation at 4 °C. APX activity was determined following the method of Liu et al. [64] by monitoring absorbance change at 290 nm. Glutathione reductase (GR) activity was measured according to the method of Chotikakham et al. [65] by detecting absorbance change at 340 nm. The enzyme activity determination methods for monodehydroascorbate reductase (MDHAR) and dehydroascorbate reductase (DHAR) were performed according to the method of Ma et al. [62] by recording absorbance change at 340 nm (DHAR) and 265 nm (MDHAR). The activities of DHAR and MDHAR are expressed as U mg^−1^ protein.

### 4.4. Measurement of Sugar Concentrations and Enzyme Activities in Sugar Metabolism

Sucrose, glucose, and fructose concentrations were assayed with HPLC–ELSD [66] and are expressed as mg·g^−1^ FW. The fructose-6-phosphate concentration was measured according to the method of Barker et al. [67], validated using authentic standards and is expressed as μg·g^−1^ FW. Sucrose phosphate synthase (SPS), sucrose synthase (SS), acid invertase (AI), and neutral invertase (NI) were extracted from kiwifruit (5 g) and assayed according to the method of Sun et al. [68]. Phosphohexose isomerase (PHI) activity was assayed according to the method of Hizukuri et al. [69]. The activity unit was defined as the changes of 0.01 absorbance at 540 nm per hour, and these enzyme activities are expressed as U g^−1^ based on protein concentration.

### 4.5. Statistical Analysis

The experimental design employed randomized biological triplication. The data are presented as the mean ± standard error (SE) and were analyzed using one-way ANOVA to identify significant differences.

## 5. Conclusions

The optimized karrikins concentration (1.20 μmol L^−1^) positively regulated kiwifruit (*Actinidia deliciosa*) redox balance and sugar metabolism by attenuating respiratory rate elevation, relative electrolyte leakage, and chromatic aberration while maintaining fruit firmness and enhancing soluble solids content. This concentration reduced ROS accumulation (O_2_^−^•, H_2_O_2_, and •OH), elevated SOD, CAT, and POD activities, and suppressed PPO activity. Simultaneously, it activated the AsA-GSH cycle through upregulated MDHAR, DHAR, GR, and APX enzyme activities, resulting in increases in GSH and AsA concentrations with decreases in GSSG and DHA concentrations. Furthermore, karrikins modulated sugar metabolism by enhancing SS (synthesis direction), SPS, and PHI activities while inhibiting SS (decomposition direction), AI, and NI, thereby promoting the accumulation of reduced ascorbate, glutathione, sucrose, and fructose-6-phosphate. As an initial exploration of karrikins in postharvest fruit preservation, this study reveals karrikins’ efficacy in enhancing kiwifruit storage quality. While current research on karrikins remains nascent in safety and edible standards, these results suggest that the proper dose of karrikins could offer a valuable method for the postharvest preservation of kiwifruit.

## Data Availability

Data are contained within the article.
